# Discovery of a notable DDT-degrading bacterium originating from insecticide-contaminated soil in Vietnam and representing a novel species

**DOI:** 10.3389/fmicb.2026.1744811

**Published:** 2026-02-13

**Authors:** Phuong Ha Vu, Linh Phuong Tran, Tung Son Vu, Trang Quynh Thi Tran, Yen Thi Nguyen, Anh Quynh Hoang, Thao Kim Nu Nguyen, Minh Hong Nguyen, Song-Gun Kim, Huyen Thanh Thi Tran, Hai The Pham

**Affiliations:** 1 GREENLAB, Center for Life Science Research (CELIFE), Faculty of Biology, VNU University of Science – Vietnam National University, Hanoi, Vietnam; 2 Department of Cell Biology, Faculty of Biology, VNU University of Science – Vietnam National University, Hanoi, Vietnam; 3 Bioresource Research Center, Phenikaa University, Hanoi, Vietnam; 4 Korean Collection for Type Cultures (KCTC)/Biological Resource Center, Korea Research Institute of Bioscience and Biotechnology (KRIBB), Daejeon, Republic of Korea; 5 Department of Microbiology, Faculty of Biology, VNU University of Science – Vietnam National University, Hanoi, Vietnam

**Keywords:** bioremediation, DDT, degradation, novel species, *Pseudomonas*, Vietnam, whole-genome sequencing

## Abstract

Dichlorodiphenyltrichloroethane (DDT), one of the earliest and most extensively used synthetic insecticides, has become a persistent organic pollutant of global concern due to its high recalcitrance and hydrophobicity (resulting in its poor water solubility and long-term accumulation in soils and sediments). In this study, 207 bacterial strains were isolated from DDT-contaminated soils and screened for their ability to grow on DDT-containing media. Among them, 10 strains exhibited remarkable tolerance and growth, with strain T006 showing the highest potential for DDT degradation. Further experiments demonstrated that T006 exhibited strong and progressive DDT-degrading activity, removing over 70% of DDT within 8 weeks of culture. Comprehensive phenotypic, chemotaxonomic, and genome-based analyses — including 16S rRNA gene and *rpoD* gene phylogeny, average nucleotide identity (ANI), and digital DNA–DNA hybridization (dDDH) — confirmed that strain T006 represents a novel species, designated as *Pseudomonas vietnamensis*. The discovery of *Pseudomonas vietnamensis* T006 expands the known diversity of DDT-degrading bacteria and provides a promising microbial resource for the development of effective bioremediation strategies targeting organochlorine pesticide–contaminated environments.

## Introduction

Dichlorodiphenyltrichloroethane (DDT) is one of the first synthetic insecticides created in the 1940s and has been widely used since then, mainly for agricultural purposes. Like other organochlorine insecticides, DDT is highly hydrophobic, i.e., poorly soluble in water, and tends to be adsorbed in soil, sediments, and sludge. This, along with its polycyclic aromatic structure containing chlorine, greatly affects the biodegradation process of DDT, making DDT very persistent in the environment (Mansouri et al., 2017). High hydrophobicity combined with long survival time in the environment allows DDT to accumulate in the fat of animals, following the biomagnification effect in the food chain (Di Landa et al., 2009). Over time, there has been increasing evidence of negative impacts of DDT exposure on human health such as endocrine disruption, inhibition of acetylcholinase enzyme activity and negative impacts on cell cycle, with the risk of causing cancer (Cohn et al., 2010; Harada et al., 2016). Therefore, degradation of DDT remaining in the environment is an urgent need. To degrade DDT, many chemical and physical methods have been applied such as landfill-isolation, reductive dechlorination using metals and electrolysis (Mosier et al., 1969; Xia et al., 2012). However, these solutions often generate secondary pollution and high treatment costs, thus the current trend focuses on biological treatment using microorganisms, which can offer complete decomposition of DDT, environmental friendliness and reasonable cost (Aparicio et al., 2017; Wang et al., 2017). Therefore, the quest for microorganisms that can effectively degrade DDT is always neccesary.

In this study, we attempted to find microorganisms from Vietnam soil that can effectively degrade DDT with the aim of obtaining ones that adapt well with environmental conditions in Vietnam. The further aim is to develop adaptive microbial inoculants to bioremediate DDT- contaminated soil, which is relatively common in many locations in Vietnam. This is important for Vietnam as till now the research on this topic is still very limited, with only 1 report on a filamentous fungus strain that can decompose DDT (Ánh and Hà, 2009). We obtained an efficiently DDT-degrading bacterial strain that turned out to be a representative of a novel *Pseudomonas* species charactersitic for Vietnam. This finding is interesting as *Pseudomonas* species are known to be metabolically versatile and thus display a great potential for application in bioremediation, including biodegradation of recalcitrant halogenated aromatic compounds such as dioxin, dichlorodiphenyltrichloroethane (DDT), gamma-hexachlorocyclohexane (lindane), etc. (Kamanavalli and Ninnekar, 2004; Nawab et al., 2003). The strain is therefore a promising candidate to develop a microbial product for effective bioremediation of DDT-contaminated soil under Vietnam conditions.

## Materials and methods

### Isolation

A 10 g soil sample previously collected from an insecticide-contaminated site in Thach That, Hanoi, Vietnam (21^o^3’21”N - 105^o^34’17″E) was mixed thoroughly with 100 mL of sterilized physiological saline in 250 mL Erlenmeyer flask, incubated for 15 min on a rotary shaker at 30 °C and 150 rpm to obtain a 10^−1^ dilution suspension. Subsequently, the dilution suspension was spread onto using a modified mineral salts medium (MSM), containing 4.0 mg L^−1^ of naphthalene, toluene or trichloroethylene (incubated for 7 days at 30 °C). The MSM contains 0.4 g L^−1^ MgSO_4_.7H_2_O, 0.002 g L^−1^ FeSO_4_.7H_2_O, 0.2 g L^−1^ K_2_HPO_4_, 0.2 g L^−1^ (NH_4_)_2_SO_4_, 0.08 g L^−1^ CaSO_4_, and 0.1% trace element solution; and its pH is adjusted to 7.0–7.2. The trace element solution contains 1 g L^−1^ FeSO_4_.7H_2_O, 0.07 g L^−1^ ZnCl, 0.1 g L^−1^ MnCl_2_.4H_2_O, 0.006 g L^−1^ H_3_BO_3_, 0.13 g L^−1^ CaCl_2_.6H_2_O, 0.002 g L^−1^ CuCl_2_.6H_2_O, 0.024 g L^−1^ NiCl_2_.6H_2_O, 0.036 g L^−1^ NaMO_4_.2H_2_O, 0.238 g L^−1^ CoCl_2_.6H_2_O; and its pH is adjusted to 7.0–7.2. Individual pure colonies were subsequently purified by three-phase streaking and cultured on Luria–Bertani (LB) medium (10 g L^−1^ peptone, 5 g L^−1^ yeast extract, 10 g L^−1^ NaCl). Once pure cultures were achieved, they were stored at 4 °C for further study.

### Determining the abilities of the strains studied to grow on DDT using the comparative shaking method

To evaluate the ability of the studied strains to grow in the presence of DDT, the following procedure was employed: Each prepared bacterial suspension (having an OD_600nm_ = 1.0–1.2) was inoculated at a seeding rate of 10% (v/v) into test tubes containing MSM medium without DDT or with 5 mg L^−1^ DDT. The cultures were incubated on a rotary shaker at 30 °C and 150 rpm for 7 days. After incubation, 200 μL from each culture was transferred into a well on a 96-well microplate, and the optical density at 600 nm (OD_600nm_) was measured using an ELISA microplate reader (Plus384, Molecular Devices, USA). The OD_600nm_ values when a strain grew without DDT and with 5 mg L^−1^ DDT were compared. A strain was considered capable of utilizing DDT for growth when its OD_600nm_ value in DDT-containing MSM significantly exceeded that in the control MSM without DDT. Such a method of determining the growth of the strains on DDT is thus called the comparative shaking method.

### Determination of DDT biodegradation efficiency using GC-FID

Preparation of bacterial suspensions: The bacterial strains of interest were first reactivated on LB agar plates to obtain fresh colonies. Selected colonies were inoculated into LB broth and incubated at 30 °C with shaking at 150 rpm. For each culture, after 24 h, the resulting cell mass was harvested by centrifugation at 6000 rpm for 15 min. The pellet was rinsed twice with MSM medium, centrifuged again to remove excess liquid, and finally resuspended in an appropriate volume of MSM medium so that the OD_600nm_ of the resulted suspension reached approximately 1.0.

To evaluate DDT degradation, 100 μL of each bacterial suspension to be tested was inoculated into a tube containing 1 mL of liquid MSM, solid MSM (1.6% agar, pH 7.0) or semi-solid MSM (0.25% agar, pH 7.0) supplemented with 30 mg L^−1^ DDT. Cultures were incubated at 30 °C, and residual DDT concentrations were analyzed after 2, 4, and 8 weeks. Thus, for each experimental case, at least 6 identical tubes were prepared, including 2 to be analyzed after 2 weeks, 2 after 4 weeks, and 2 after 8 weeks.

At each sampling point, 1 mL of chloroform was added to the culture tube to be analyzed and left to stand for 14 h to allow DDT extraction. The chloroform layer was then collected and its DDT concentration determined using a gas chromatography system (Agilent 7,890) equipped with an HP-5 glass capillary column (30 m × 0.32 mm ID, 0.25 μm film thickness). Nitrogen was used as the carrier gas at a flow rate of 1.2 mL min^−1^. The injector was operated in split mode (1:10), with an injection volume of 1 μL. The GC program was set as follows: injector temperature 250 °C; oven temperature initially 170 °C (held for 5 min), increased at 35 °C min^−1^ to 275 °C (held for 5 min); detector (FID) temperature 275 °C. Under these conditions, the DDT peak was detected at approximately 10 min. The concentration of DDT in each sample was determined based on a calibration curve established from standard samples, which were 1 mL aliquots of MSM broth containing 10, 30, and 50 mg L^−1^ DDT, for liquid medium samples, or 1 cm^3^ MSM agar pieces containing 10, 30, and 50 mg L^−1^ DDT, for solid or semi-solid medium samples.

### Morphological observations and phenotypic characterization

Cell morphology of the strain of interest was observed after growth for 24 h on LB medium agar at 37 °C by using a scanning electron microscope (FE-SEM S-4800, Hitachi, Japan) at National Institute of Hygiene and Epidemology (Vietnam). The ability of the bacteria to produce pigments and colony texture were tested on King’s B agar (King et al., 1954). Gram staining was performed using the standard method (Bartholomew and Mittwer, 1952). The temperature range for growth was measured in LB broth after incubation at 10, 15, 20, 25, 30 and 40 °C, with shaking at 200 rpm. The pH range for growth (pH 5.0–11.0, with intervals of 2 pH unit) was determined after incubation at 30 °C, 200 rpm in LB broth. Salt tolerance for growth was examined in LB broth with different NaCl concentrations (0–5%, w/v, with intervals of 1.0%) at 30 °C, 200 rpm. Growth was evaluated by spectrophotometric measurement of the optical density of cells at 600 nm (OD_600nm_). Other enzyme activities and substrate utilization were determined using the API 20NE kits (bioMérieux, France) according to the manufacturers’ instructions.

Fatty acid composition was analysed by gas chromatography (on an Agilent 8,890 system) following standard procedures at Korean Collection for Type Cultures (KCTC) (Korea). The strains of interest were cultivated on TSA medium for 4 days at 28 °C before analysis. The major fatty acids detected in T006 were C_16:0_, summed feature 3 (C16:1ω6c and/or C16:1ω7c) and summed feature 8 (C18:1 ω7c and/or C18:1ω6c).

### DNA extraction

The cell suspension was incubated overnight in LB medium at 30 °C with shaking at 150 rpm (OD_600nm_ = 1.0). Genomic DNA was then extracted from the harvested biomass using a DNeasy Blood and Tissue kit (Qiagen, USA), following the manufacturer’s instructions.

### Phylogenetic analysis

Primary phylogenetic analysis was done on the 16S rRNA gene sequence. The 16S rRNA gene fragment of the strain of interest was first amplified from its genomic DNA using the universal primer pair 27F (forward primer, 5’-AGAGTTTGATCCTGGCTCAG-3′) and 1492R (reverse primer, 5’-TACCTTGTTACGACTT-3′) (Heuer et al., 1997). The PCR program included an initial denaturation step at 96 °C for 3 min, followed by 35 cycles consisting of denaturation at 95 °C for 45 s, annealing at 55 °C for 45 s, and extension at 95 °C for 2 min. A final extension was performed at 68 °C for 2 min. A more thorough phylogenetic analysis was done on the *Pseudomonas*-specific *rpoD* gene sequence. A *rpoD* gene fragment of the strain of interest was amplified from its genomic DNA using the primers PPSEG30F (5’-ATYGAAATCGCCAARCG-3′) and PPSEG790R (5’-CGGTTGATKTCCTTGA-3′) (Girard et al., 2020). The thermal cycle began with 3 min of denaturation at 95 °C, followed by 35 cycles of 15 s at 95 °C (denaturation), 30s at 50 °C (annealing), and 15 s at 68 °C (extension), ending with a final 2 min extension at 68 °C. The 16S rRNA gene and *rpoD* gene PCR products were sent for sequencing by Genlab (Vietnam) using the standard Sanger method.

The phylogenetic identification of the strain of interest was determined by comparing its 16S rRNA gene sequence and *rpoD* gene sequence with those of other type strains obtained from the National Centre for Biotechnology Information (NCBI) GenBank database and the EzTaxon-e database (Yoon et al., 2017a). The sequences of these genes were aligned using the ClustalX program (Kumar et al., 2018). To calculate evolutionary distances, Kimura’s two-parameter model (Kimura, 1979) was applied, and a phylogenetic tree was then constructed using neighbour-joining (NJ) algorithm (Trees, 1987) with bootstrap values based on 1000 replications.

### Whole genome sequencing and analysis

Whole genome sequencing was carried out by LOBI (Hanoi, Vietnam) using a BGI sequencing system (BGISEQ-500). The genome was subsequently assembled using SPAdes after decontamination filtering, and contigs shorter than 500 bp were discarded. *De novo* assembly of the genome sequences was performed using Prokka.

The whole genome data of any two strains of interest were compared and their average nucleotide identity (ANI) values and digital DNA–DNA hybridization (dDDH) values were calculated using the OrthoANIu algorithm^1^ (Yoon et al., 2017b), and the Genome-to-Genome Distance Calculator (GGDC) version 2.1^2^ (Meier-Kolthoff et al., 2013), respectively.

Annotation of the genome of the strain of interest was carried using the Kyoto Encyclopedia of Genes and Genomes (KEGG) as follows: (i) first, KEGG Orthology (KO) identifiers corresponding to the genes in the genome were retrieved and compiled; (ii) second, the compiled KO dataset was subsequently uploaded to KEGG server by using the KEGG mapper – reconstruction tool to generate the comprehensive metabolic pathway profile of the strain; (iii) third, the enzymes of the strain were annotated by the KEGG database and placed on “map01120: Microbial metabolism in diverse environments”, as this is the only metabolic map that involves DDT. Based on that, the DDT metabolizing potential of the strain could be predicted.

### Data analysis

Unless otherwise stated, the experiments were performed in triplicates and the reported data were average values and standard deviations calculated using standard Microsoft Excel algorithms. Significance of data differences between different experimental cases was evaluated using the basic *t*-test.

## Results

### Isolation

From the collected soil samples, 207 bacterial strains were isolated. They were the isolates that could grow well in mineral salt medium supplemented with naphthalene, toluene or trichloroethylene as the carbon source. These compounds are to some extent structurally similar to DDT in that they are aromatic or chloride-containing substances. Therefore, the obtained strains have the great potential to also metabolize DDT.

### Preliminary screening for strains growing on DDT

**Figure 1:**
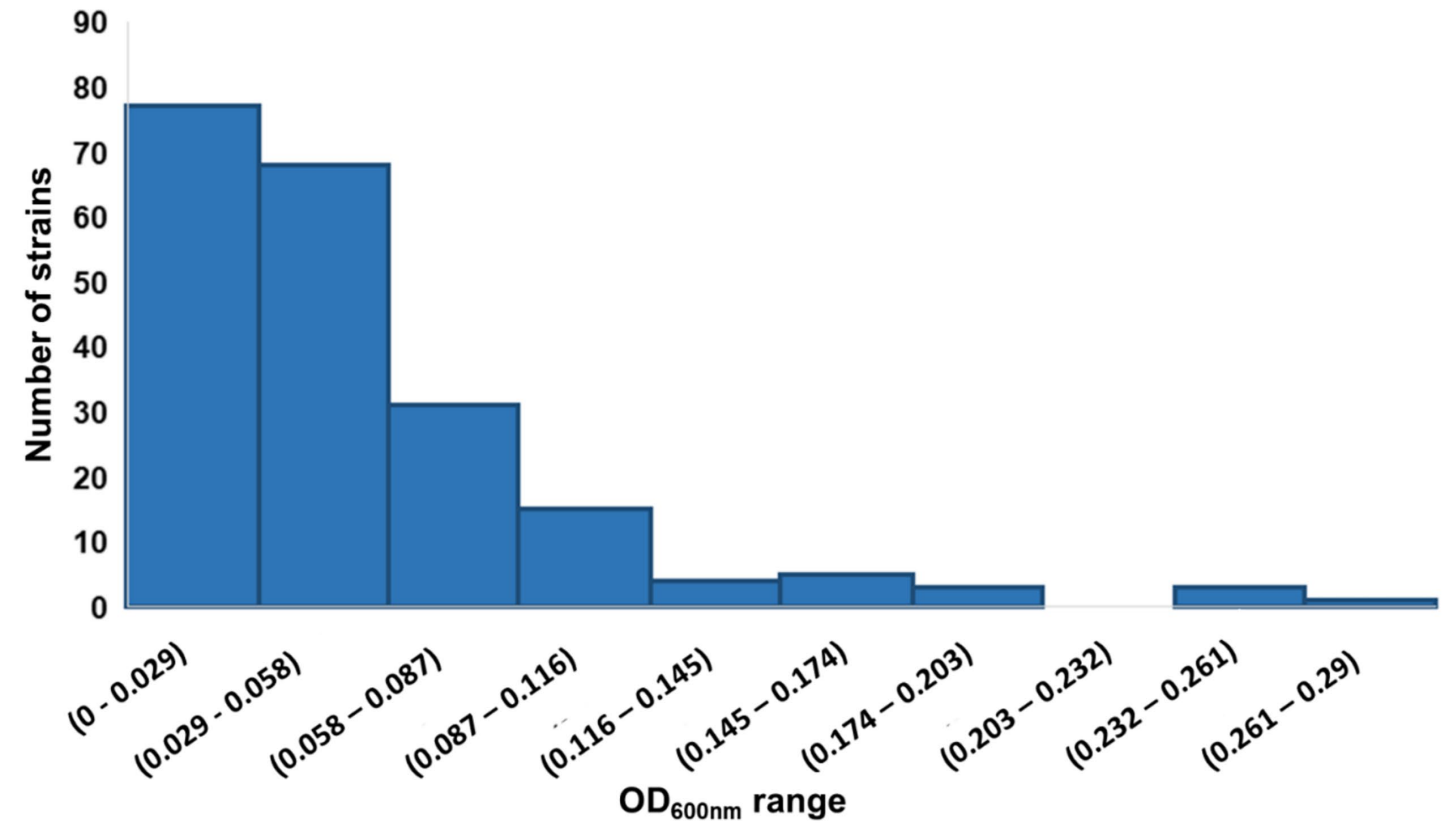
Distribution of 207 isolated strains according to their growth (indicated by OD_600nm_ values) in the MSM medium containing 5 mg L^−1^ DDT.

The isolated strains were examined for their ability to grow in MSM medium with DDT as the sole carbon source using the comparative shaking incubation method. Previously, only a few microorganisms were reported to be able to grow in minimal medium with DDT as the sole carbon source, such as *Stenotrophomonas* sp. DDT-1 isolated by Pan et al. (2016). The strains with their OD_600nm_ values in MSM medium supplemented with 5 mg L^−1^ DDT higher than in those in the medium without DDT were considered to have the potential to use DDT as a nutrient source for growth (Figure 1).

By that principle, out of the 207 bacterial strains, 10 were selected, displaying outstanding growth on the medium containing DDT (Table 1). In particular, strain T006 displayed the best growth and thus we decided to further investigate this strain in terms of DDT degradability.

**Table 1:** Top 10 isolated strains exhibiting the most robust growth in MSM medium containing 5 mg L^−1^ DDT and their respective growth-indicating OD_600nm_ values.

No.	Name	MSM	MSM + 5 mg L^−1^ DDT
1	T006	0.042 ± 0.006	0.275 ± 0.005
2	T044	0.055 ± 0.004	0.247 ± 0.008
3	T036	0.047 ± 0.005	0.242 ± 0.004
4	T033	0.033 ± 0.005	0.241 ± 0.008
5	Y005	0.057 ± 0.007	0.198 ± 0.007
6	T012	0.110 ± 0.006	0.188 ± 0.006
7	Y007	0.044 ± 0.008	0.174 ± 0.005
8	T026	0.076 ± 0.003	0.164 ± 0.009
9	T019	0.048 ± 0.009	0.158 ± 0.007
10	A003	0.092 ± 0.004	0.155 ± 0.005

### DDT biodegradation capability of T006

In order to accurately evaluate the DDT degradation capability of T006, changes of DDT concentration were monitored when this strain was grown in liquid, semi-solid and solid MSM media containing 30 mg L^−1^ DDT as the only carbon source. As shown in Table 2, the remaining DDT concentrations decreased significantly with time in all three tested medium forms (liquid, soil, and semi-solid), while did not change significantly in the uninoculated controls. In the liquid medium experiment, ~39% DDT was removed after 2 weeks and ~58% DDT was removed after 8 weeks of incubation. An additional kinetic investigation showed that DDT concentration decreased in a decaying trend and DDT degradation was strongly associated with the growth of T006 (Supplementary Figure S1). Furthermore, the sole intermediate produced was DDE but the concentration of this compound also decreased further with time, while all the concentrations of DDT and possible metabolites already decreased to low levels after 2 weeks (Supplementary Figure S2). These observations suggest that DDT degradation by T006 might not stop at several transformation steps but probably lead to complete minineralization. Similarly, in the semi-solid medium experiment, only ~36% DDT was removed after 2 weeks but up to ~71% was removed after 8 weeks. Surprisingly, in the solid medium experiment, already ~68% DDT was removed after 2 weeks but after that DDT did not seem to be removed significantly further. In general, considering that in the control experiment DDT level showed almost no decrease, the presence of strain T006 is apparently associated to the effective degradation of DDT, which demonstrates the excellent DDT-degrading capability of the strain.

**Table 2:** Remaining DDT (% vs. the initial concentration) in different experiments culturing T006 in different forms of MSM medium containing 30 mg L^−1^ DDT.

Media		2 weeks	4 weeks	8 weeks
Liquid	Control	100.30 ± 0.05	93.57 ± 1.18	93.27 ± 3.56
	T006	61.05 ± 0.20	52.28 ± 3.14	42.47 ± 1.26
Solid	Control	96.91 ± 7.21	86.08 ± 6.47	83.50 ± 3.52
	T006	32.24 ± 0.33	24.19 ± 2.41	34.43 ± 6.02
Semi-solid	Control	99.91 ± 2.55	99.59 ± 1.28	103.88 ± 1.65
	T006	64.42 ± 2.79	50.49 ± 0.34	28.91 ± 3.23

In view of the notable DDT-degrading performance of strain T006, further characterization was subsequently carried out to taxonomically identify the strain.

### Morphological features of strain T006

**Figure 2:**
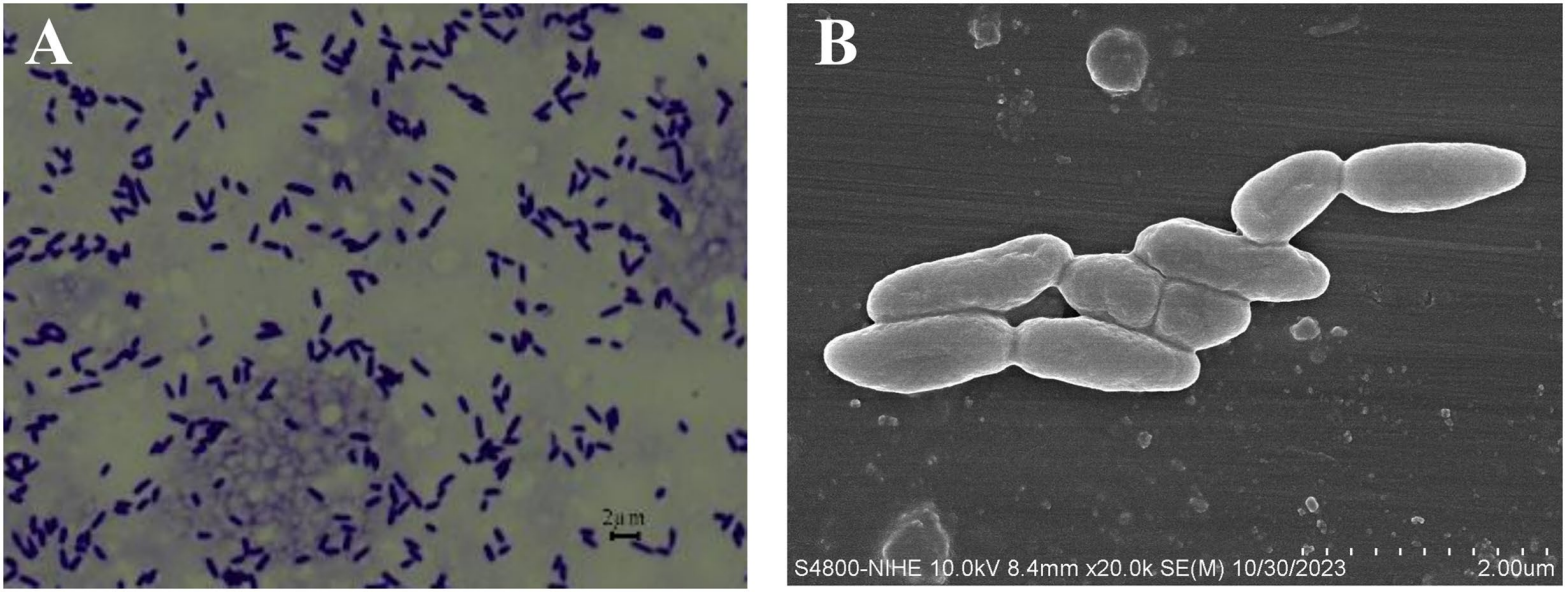
Cell morphology of strain T006. **(A)** Cells observed with light microscope; **(B)** Cells observed with SEM.

The cells of this strain are Gram-negative and rod-shaped (0.6–0.9 μm × 0.9–1.9 μm) (Figure 2). Colonies were round, convex, smooth and did not produce fluorescent pigments when cultured on King’s B agar 2 days at 25 °C. The strain could grow at pH 5.0–9.0 and in the presence of 0–5% (w/v) NaCl at 10–40 °C (optimally at 30 °C). T006 was capable of assimilating D-glucose, potassium gluconate, capric acid, adipic acid, malic acid, trisodium citrate, and phenylacetic acid; it could also reduce nitrates and hydrolyze urea, as well as exhibit arginine dihydrolase activity.

### Phylogenetic analysis

**Figure 3:**
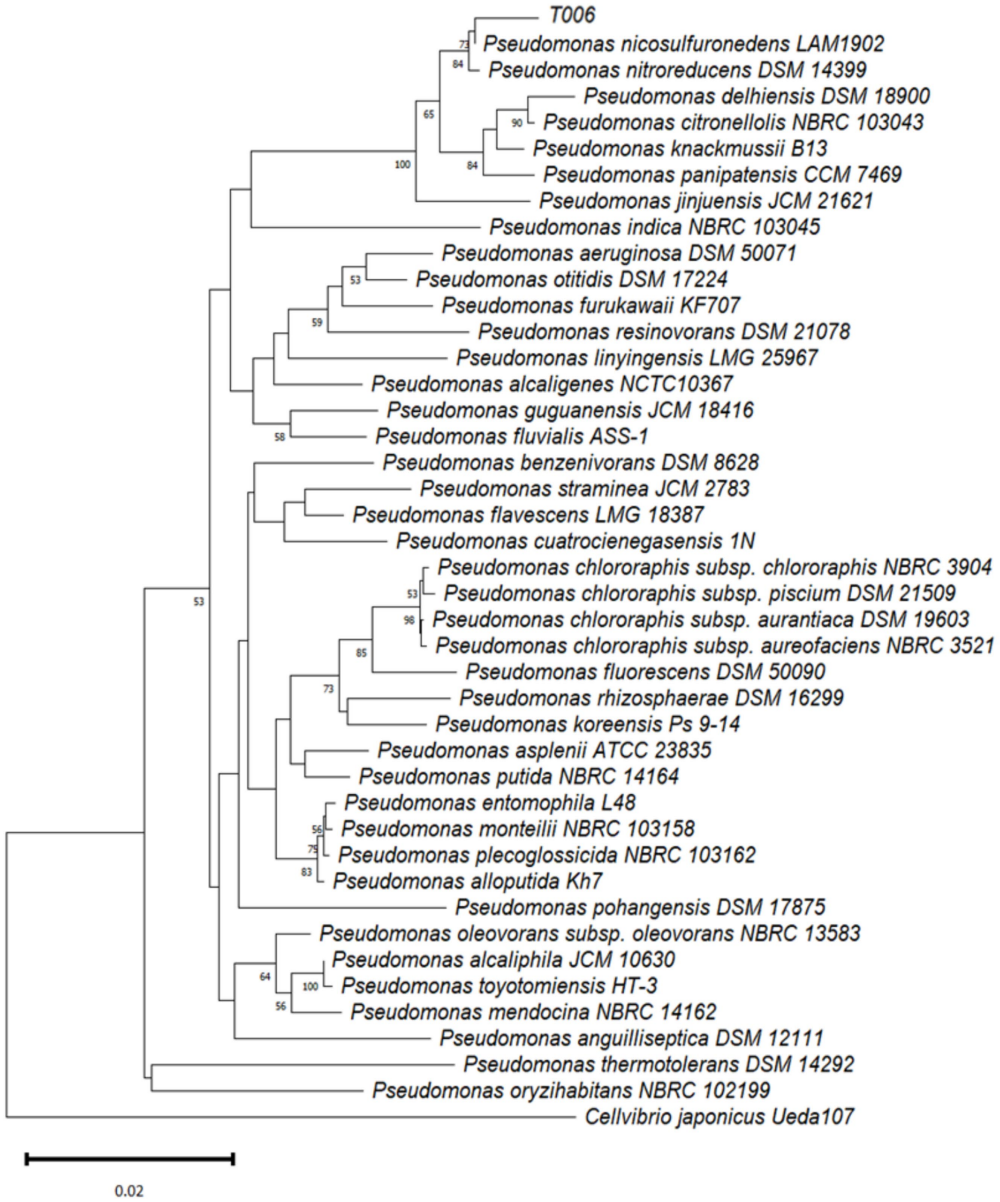
Neighbor-joining (NJ) tree based on 16S rRNA gene sequence comparison, showing the taxonomic position of strain T006 within the genus *Pseudomonas*. The NJ tree was constructed using Kimura’s two-parameter model with bootstrap values based on 1,000 replications.

The 16S rRNA gene sequence (GenBank accession number OR484872) of T006 was the most homologous to those of *Pseudomonas* species, especially *Pseudomonas nicosulfuronedens* (99.39%) and *Pseudomonas nitroreducens* (99.11%) (Figure 3). Thus, based on this phylogenetic analysis result, T006 is proposed to belong to the *Pseudomonas* group and was the closest to *Pseudomonas nicosulfuronedens* and *Pseudomonas nitroreducens*.

**Figure 4:**
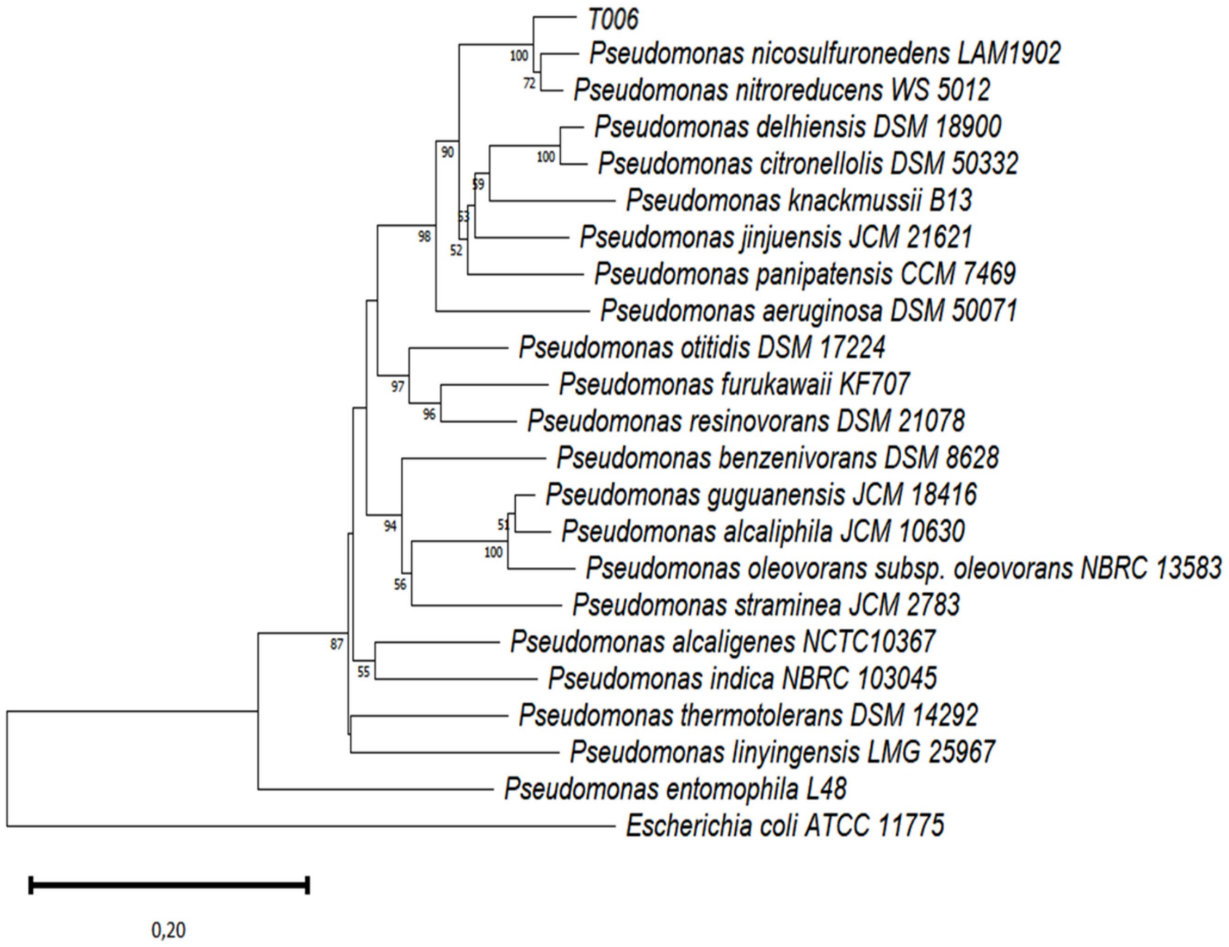
Neighbor-joining (NJ) tree based on *rpoD* gene sequences, showing the taxonomic position of strain T006 within the genus *Pseudomonas*. The NJ tree was constructed using Kimura’s two-parameter model with bootstrap values based on 1,000 replications.

However, based on 16S rRNA gene sequences, it is now not feasible to identify bacteria, especially Gram-negative ones, to the species level (Girard et al., 2020). Therefore, using similar procedures, we also amplified the *rpoD* gene of T006 and carried out another phylogenetic analysis by comparing the obtained sequence (GenBank accession number OR501201) with those of the *rpoD* genes of other type strains of the *Pseudomonas* group, taken from the NCBI GenBank database. The results confirmed that T006 was the closest to two species, *Pseudomonas nitroreducens* (93.99%) and *Pseudomonas nicosulfuronedens* (93.30%) (Figure 4). However, as the cut-off value for the *rpoD* gene similarity to determine whether two bacterial strains belong to the same species is 98% (Girard et al., 2020), T006 might belong to an as-yet-undescribed species of *Pseudomononas.*

### Genomic analysis

Whole-genome sequencing of T006 was conducted and good quality data were obtained, as indicated by the quality control results, with an average quality score above 30 for all sequencing segments. The genome sequence data of T006 were obtained and deposited in the NCBI database under the SRA accession number SRX21629081 and the biosample accession number SAMN37187830.

After assembled using SPAdes, the final assembly consisted of 27 contigs with a total genome size of 6,472,987 bp, the largest contig length of 857,778 bp, GC content of 65.79%, and an N50 value of 558,527 bp. These parameters indicate a high-quality draft genome suitable for downstream functional analysis. Genome annotation further identified 5,869 predicted protein-coding sequences (CDSs), together with 5 rRNA genes, 51 tRNA genes and one tmRNA. Assessment of genome completeness using BUSCO indicated a completeness of 99.6%, confirming that the assembled genome of strain T006 is nearly complete.

After *de novo* assembly, the whole genome sequence of T006 was compared with those of 20 type strains (obtained from GenBank), and their ANI and dDDH values were determined (Table 3). The ANI values between T006 and the other species ranged from 77.88–91.93% (Table 3), and with the intra-species ANI cut-off value set at 95%, we again confirm that T006 does not belong to any other species. Moreover, the dDDH values between T006 and the other species ranged from 22.00–46.6% (Table 3), and with the intra-species DDH cut-off value set at 70%, we hypothesize that T006 represents a novel species of the genus *Pseudomonas*.

**Table 3:** Average nucleotide identity (ANI), and digital DNA–DNA hybridization (dDDH) values between the genome sequence of strain T006 and those of 21 type strains of closely related *Pseudomonas* species.

Species	OrthoANI (%)*	dDDH (%)^†^
		Fomula 2
*Pseudomonas nicosulfuronedens* LAM1902 ^(T)^	91.93	46.60
*Pseudomonas nitroreducens* DSM 14399 ^(T)^	90.86	42.50
*Pseudomonas delhiensis* DSM 18900 ^(T)^	84.48	27.80
*Pseudomonas citronellolis* NBRC 103043 ^(T)^	84.40	27.80
*Pseudomonas jinjuensis* JCM 21621 ^(T)^	83.61	27.10
*Pseudomonas knackmussii* B13 ^(T)^	83.47	26.50
*Pseudomonas panipatensis* CCM 7469 ^(T)^	82.97	26.10
*Pseudomonas aeruginosa* DSM 50071 ^(T)^	81.09	24.00
*Pseudomonas otitidis* DSM 17224 ^(T)^	79.71	22.70
*Pseudomonas thermotolerans* DSM 14292 ^(T)^	79.60	22.50
*Pseudomonas furukawaii* KF707 ^(T)^	79.41	22.70
*Pseudomonas alcaligenes* NCTC10367 ^(T)^	79.31	22.60
*Pseudomonas benzenivorans* DSM 8628 ^(T)^	78.89	22.50
*Pseudomonas linyingensis* LMG 25967 ^(T)^	78.86	22.50
*Pseudomonas resinovorans* DSM 21078 ^(T)^	78.69	22.20
*Pseudomonas indica* NBRC 103045 ^(T)^	78.61	21.90
*Pseudomonas guguanensis* JCM 18416 ^(T)^	78.58	22.10
*Pseudomonas oleovorans subsp. oleovorans* NBRC 13583 ^(T)^	78.37	22.10
*Pseudomonas alcaliphila* JCM 10630 ^(T)^	77.90	21.70
*Pseudomonas entomophila* L48 ^(T)^	77.88	22.00
*Pseudomonas straminea* JCM 2783 ^(T)^	77.82	21.60

^*^The ANI values were calculated using the software OAT. ^†^The dDDH values were calculated using the GGDC (http://ggdc.dsmz.de/distcalc2.php).

### Phenotypic characteristics comparison

The phenotypic characteristics of T006 were compared with the two type strains that showed the highest ANI scores against the T006 genome. The differences among them (summarized in Table 4) showed that T006 could be distinguished from its genetically closest species, *Pseudomonas nicosulfuronedens* and *Pseudomonas nitroreducens,* by its ability to hydrolyze urea and to grow in the prsence of 5% (w/v) NaCl, as confirmed by conventional methods.

**Table 4:** Comparison of phenotypic characteristics of strain T006 and the type strains of the most closely related *Pseudomonas* species.

Characteristics	Strain 1	Strain 2	Strain 3
Temperature range for growth (°C)	15–40	20–35	Optimum 30
NaCl concentration range for growth (%, w/v)	0–5	0–4	0–4
pH range for growth	5–9	5–10	Optimum pH 8
Fluorescent pigment production on King’s B medium	−	ND	−
Assimilation of			
D-glucose	+	+	+
L-arabinose	−	−	−
D-mannose	−	−	−
D-mannitol	−	−	−
N-acetyl-glucosamine	−	−	−
D-maltose	−	−	−
Potassium gluconate	+	+	+
Capric acid	+	+	+
Adipic acid	+	+	+
Malic acid	+	+	+
Trisodium citrate	+	+	+
Phenylacetic acid	+	+	+
Reduction of			
Nitrates	+	+	+
Hydrolysis of			
Urea	+	−	−
Esculin	−	ND	−
Gelatin	−	-	−
Indole production	-	−	−
Fermentation of D-glucose	−	-	−
Arginine dihydrolase	+	+	+
β-Galatosidase	−	ND	−

Strain 1: T006; strain 2: *Pseudomonas nicosulfuronedens* LAM1902 ^(T)^; strain 3: *Pseudomonas nitroreducens* DSM 14399 ^(T)^. +: Positive; −: negative; w: weakly positive; ND: not done. Data for the other type strains were obtained from references (Li et al., 2021).

### Fatty acid profile

T006 was also found to have three cellular fatty acids generally detected for the genus *Pseudomonas*: C_10:0_ 3OH; C_12:0_; and C _12:0_ 3OH (Table 5). However, its cellular fatty acid profile was significantly different from those of the two closely related species (Table 5), which corroborates the hypothesis that the strain does not belong to those two species.

**Table 5:** Cellular fatty acid compositions of strain T006 and two closely related *Pseudomonas* species.

Fatty acids	Strain 1	Strain 2	Strain 3
C_10:0_ 3OH	3.74	4.85	2.8
C_12:0_	2.18	1.90	3.15
C_11:0_ iso 3OH	0.19		
C_12:0_ 2OH	6.31	7.65	6.0
C _12:0_ 3OH			
C _14:0_	0.48	1.31	ND
C _15:0_ iso	0.18		
C _15:0_	ND	ND	
C _16:1_ w5c	0.17	0.16	
C _16:0_	21.47	24.48	25.2
C _17:0_ iso	0.47		
C _17:1_ w8c	0.29		
C _17:0_ cyclo	6.66	7.44	14.1
C _17:0_	0.28	0.23	
C _18:0_	0.77	0.58	
C _18:1_ w7c 11-methyl	0.12		
C _19:0_ cyclo w8c	5.90	3.50	5.2
C _20:2_ w6,9c	0.15		
Summed Feature 3	13.49	13.67	7.8
Summed Feature 8	32.20	28.38	

Strain 1: T006; strain 2: *Pseudomonas nicosulfuronedens* LAM1902^(T)^; strain 3: *Pseudomonas nitroreducens* DSM 14314^(T)^; ND: Not detected. Summed feature 3 contains one or more of the fatty acids C_16:1ω6c_ and C_16:1ω77_. Summed feature 8 contains one or more of the fatty acids C_18:1 w6c._

Collectively, all phenotypic, phylogenetic, genomic and biochemical taxonomic analyses results suggest that strain T006 should be proposed to belong to a novel *Pseudomonas* species, closely related to *Pseudomonas nicosulfuronedens* and *Pseudomonas nitroreducens.* Considering its origin, we propose the name of the novel species as *Pseudomonas vietnamensis*, with T006 being its type strain. The strain was deposited in Phenikaa University Gene Resource Center as PU50001, and in Korean Collection for Type Cultures (KCTC) (Korea) as KCTC8593.

### Metabolic exploitation of the novel species *Pseudomonas vietnamensis*

**Figure 5:**
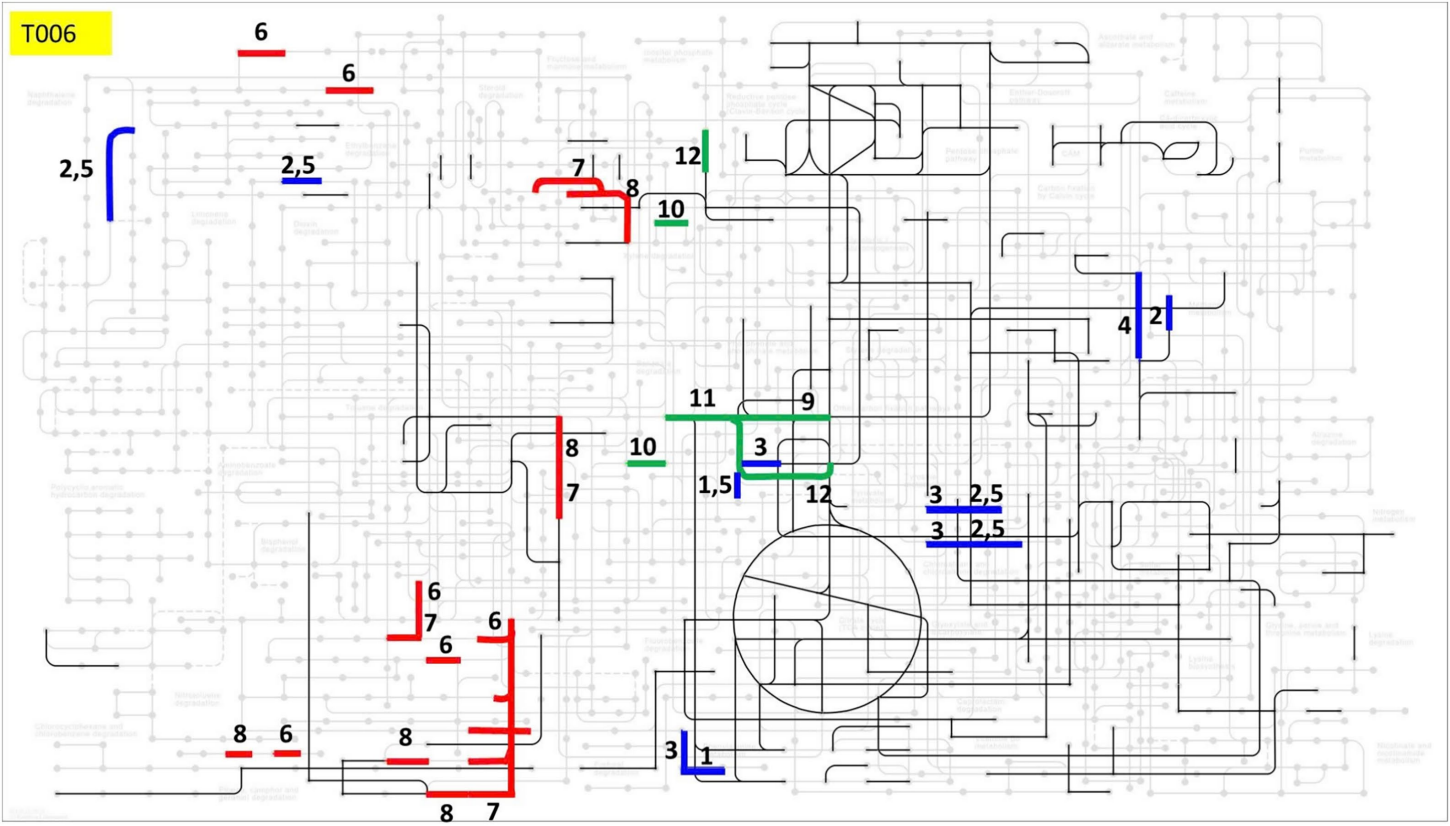
Theoretical metabolic map of T006. Bold lines indicate pathways possibly involved in the metabolism of chlorinated compounds (e.g., DDT), with different colors denoting different groups of pathways: Blue: Chloroalkane and chloroalkene degradation; Red: Chlorocyclohexane and chlorobenzene degradation pathways; Green: Dioxin degradation pathways; Black: The common pathways. (1 – Alcohol dehydrogenase; 2 – S-(hydroxymethyl) glutathione dehydrogenase; 3 – Aldehyde dehydrogenase; 4 – Glutathione-independent formaldehyde dehydrogenase; 5 – Alcohol dehydrogenase, propanol-preferring; 6 – Carboxymethylenebutenolidase; 7 – Muconate cycloisomerase; 8 – Catechol 1,2-dioxygenase; 9–4-hydroxy 2-oxovalerate aldolase; 10–4-oxalocrotonate tautomerase; 11–2-keto-4-pentenoate hydratase; 12 – Acetaldehyde dehydrogenase).

As shown in Figure 5, in addition to the basic metabolic pathways, *Pseudomonas vietnamensis* T006 has a number of additional specific enzymes possibly involved in the metabolism of chlorinated compounds. These enzymes include alcohol dehydrogenase, aldehyde dehydrogenase, glutathione-independent formaldehyde dehydrogenase, carboxymethylenebutenolidase, muconate cycloisomerase, catechol 1,2-dioxygenase. Indeed, strain T006 clearly displayed its oxidative enzyme activity and its dehalogenation activity (see Supplementary material). Our hypothesis is that T006 degrades DDT solely through oxidation-based pathways (Mansouri et al., 2017), starting with oxygenation and subsequently cleaving the aromatic ring with the enzymes of the dioxin degradation pathway (see green lines in Figure 5) and also dehydrogenases. We should not exclude the possibility that T006 may also have its own distinct pathways, which need to be investigated in further studies. All the information mentioned above demonstrates the great capability of *Pseudomonas vietnamensis* T006 as a DDT biodegrader. Considering also its Vietnamese origin, T006 appears as a very potential candidate for use in developing efficient microbial solutions to clean up the toxic insecticide DDT remaining in soil in Vietnam.

Several *Pseudomonas* strains have previously been reported to transform or degrade DDT and its metabolites. For example, *Pseudomonas acidovorans* M3GY was shown to cometabolically transform DDT to DDD when grown on biphenyl (Hay and Focht, 1998), although no degradation rate was reported. An immobilized *Pseudomonas fluorescens* strain was able to degrade DDT with efficiencies ranging from 55 to 99% at high pesticide concentrations (Santacruz et al., 2005). In an earlier study, a *Pseudomonas putida* strain was found to extensively metabolize DDT with an overall degradation efficiency of approximately 73% (Pfaender and Alexander, 1972). The DDT biodegradation efficiency of strain T006 in the present study, which already reached >60% after 2 weeks under certain circumstances and >70% after 8 weeks, is therefore comparable to, or even in some cases higher than, those reported for other *Pseudomonas* species. Beyond *Pseudomonas* species, compared to other known DDT-metabolizing bacteria, *P. vietnamensis* T006 can be ranked among the top degraders (Supplementary Table S3). This fact supports the high degradation potential of this strain.

*Pseudomonas* species are well known for their great importance in bioremediation, especially for biodegradation of persistent organochloride pollutants such as DDT, dioxin, lindane, etc. (Kamanavalli and Ninnekar, 2004). The key reason for that is the capabilities of *Pseudomonas* bacteria to function robustly and flexibly in different environmental conditions, owing to their mebolic diversity and versatility, i.e., they possess numerous enzymes that enable them to metabolize various substrates (Nawab et al., 2003). *Pseudomonas* species are also reported to have many interactions with other microorganisms in various biodegradation scenarios, as demonstrated by several co-culture studies (Mansouri et al., 2017). Therefore, the discovery of a novel DDT-degrading *Pseudomonas* species originating from Vietnam soil is particularly meaningful, as this species can serve as a new promising gene resource to be exploited for bioremediation of DDT-contaminated soils. This is also important for Vietnam, given the urge to bioremediate polluted soil that is still commonly existing in the country while there has been almost no report on potential biocatalysts, including *Pseudomonas* species, that can address that urge. As mentioned, strain T006, a representative of the novel *Pseudomonas vietnamensis* species, not only displayed a strong DDT-degrading activity but also possibly possessed diverse relevant metabolic pathways. Furthermore, this species potentially has the advantages to survive and degrade DDT well in soil under field conditions in Vietnam, as demonstrated by the wide adaptation ranges of T006 (temperature range of 15-40 °C, pH range of 5–9, salinity range of 0–5% – see Table 4) and its consistent degradation performance in semi-solid and solid media, which are relatively close to soil in structure. Thus apparently, the great potential of this novel species has already been displayed.

In summary, strain T006, a native bacterium isolated from DDT-contaminated soil in Hanoi, exhibited excellent DDT-degrading activity, with the removal efficiency achieving approximately 30–70% within the first 2 weeks and over 70% within eight weeks, making it ranking among the top DDT degraders. The strain also displayed advantages for field applications owing to its adaptability to wide ranges of environmental conditions. Genomic and phenotypic analyses demonstrate that T006 represents an undescribed *Pseudomonas* species, which is proposed to be designated as *Pseudomonas vietnamensis*. As an indigenous microorganism with strong biodegradation capacity, strain T006 holds a remarkable promise for the development of locally adapted bioformulations to remediate DDT-polluted soils in Vietnam, offering a sustainable and environmentally friendly strategy for managing persistent organochlorine pesticide contamination.

## Data Availability

The data presented in this study are publicly available. The data can be found at: https://www.ncbi.nlm.nih.gov/genbank/, accession numbers OR501201, SRX21629081, and OR484872.

## Footnotes

1
http://www.ezbiocloud.net/tools/ani

2
http://ggdc.dsmz.de
