# Supplementary material for: Discovery of a notable DDT-degrading bacterium originating from insecticide-contaminated soil in Vietnam and representing a novel species
Source: Front Microbiol. 2026 Feb 13;17:1744811. doi: 10.3389/fmicb.2026.1744811 (PMC12946071; doi:10.3389/fmicb.2026.1744811)
Supplement: Supplementary file 1 [file Table_1.docx]

**Supplemental for “Discovery of a notable DDT-degrading bacterium originating from insecticide-contaminated soil in Vietnam and representing a novel species” by Vu et al.**

**Determination of Chloride-Releasing Enzyme Activity by T006**

The method for evaluating chloride-releasing enzyme activity was adapted from Bergmann and Sanik (1957) with minor modifications (Bergmann, J. & Sanik, J., *Analytical Chemistry*, 29(2), 241–243):

Briefly, 10% (v/v) of the bacterial suspension was inoculated into test tubes containing minimal salt mdieum (MSM) and MSM supplemented with 30 ppm DDT, respectively, and incubated for 7 days at 30 °C with shaking at 150 rpm. After incubation, 200 µL of the culture supernatant was transferred to each well of a 96-well microplate, followed by the addition of 20 µL of 0.25 M ferric ammonium sulfate [Fe(NH₄)(SO₄)₂·12H₂O] in 9 M nitric acid and 20 µL of a saturated mercuric thiocyanate solution in ethanol. The mixtures were gently mixed and the color development was visually monitored, in which the appearance of a yellow color indicated the presence of free chloride ions.

After 10 min of reaction, the absorbance was measured at 460 nm using a microplate spectrophotometer. A significant difference in the absorbance at 460 nm between the analytical sample and the background medium (MSM) indicates a significant dehalogenation activity.

The result (Table S1) clearly indicates that T006 displayed dehalogenation activity.

Table S1. Measurement of Chloride-Releasing Enzyme Activity by T006

|  | **OD (460nm)** | | |
| --- | --- | --- | --- |
|  | **DDT30** | **MSM** | **Difference** |
| **Control** | 0.0451 ± 0.0010 | 0.0456 ± 0.0029 | -0.0005 |
| **T006** | 0.3773 ± 0.0060 | 0.2282 ± 0.0054 | 0.1491 |

**(DDT30**: MSM supplemented with 30 ppm DDT; **MSM:**  Minimal salt medium only )

**Determination of Oxidative Enzyme Activity**

The method for evaluating oxidative enzyme activity was adapted from Kamanavalli and Ninnekar (2004) with minor modifications (Kamanavalli, C. M. & Ninnekar, H. Z., *Current Microbiology*, 48, 10–13).

Briefly, the target strain was first reactivated on LB agar plates to obtain fresh colonies. Single colonies were inoculated into LB broth supplemented with 10 ppm DDT and incubated at 30 °C with shaking at 150 rpm for 48 h. The cultures were then subjected to sonication for 5 min, followed by centrifugation at 15,000 × g for 45 min at 4 °C.

After centrifugation, 5 mL of the supernatant was transferred to a new tube and supplemented with a DDT stock solution to obtain a final concentration of 1 µmol DDT. Dissolved oxygen (DO) was subsequently measured at defined time intervals to monitor oxygen consumption in the reaction mixture, thereby determining oxidative enzyme activity. A decrease in DO concentration was interpreted as evidence of oxidative enzymatic activity.

The result (Table S2) shows that T006 displayed a strong oxidative activity.

Table S2. Measurement of Oxidative Enzyme Activity by T006

|  | **DO (mg/L)** | | | |
| --- | --- | --- | --- | --- |
|  | **0 min** | **90 min** | **180 min** | **270 min** |
| **Control** | 8.45 | 8.64 | 8.16 | 8.27 |
| **T006** | 3.82 | 3.79 | 0.21 | 0.07 |

**DDT degradation vs. growth kinetics**:

To evaluate DDT degradation versus growth, 200 µL of the bacterial suspension of T006 was inoculated into a tube containing 1 mL of liquid MSM, solid MSM (1.6% agar, pH 7.0) or semi-solid MSM (0.25% agar, pH 7.0) supplemented with 30 mg L⁻¹ DDT. (The seeding rate therefore doubled that used the previous experiments explained in the main manuscript). The culture was incubated at 30°C, and residual DDT concentrations were analyzed after 2 days, 7 days (1 week) and 14 days (2 weeks), while the cell density (OD at 600nm) of the culture was monitored bi-daily.

The results (Figure S1) showed that DDT concentration in the culture gradually decreased and the decrease is well correlated with the growth of T006.


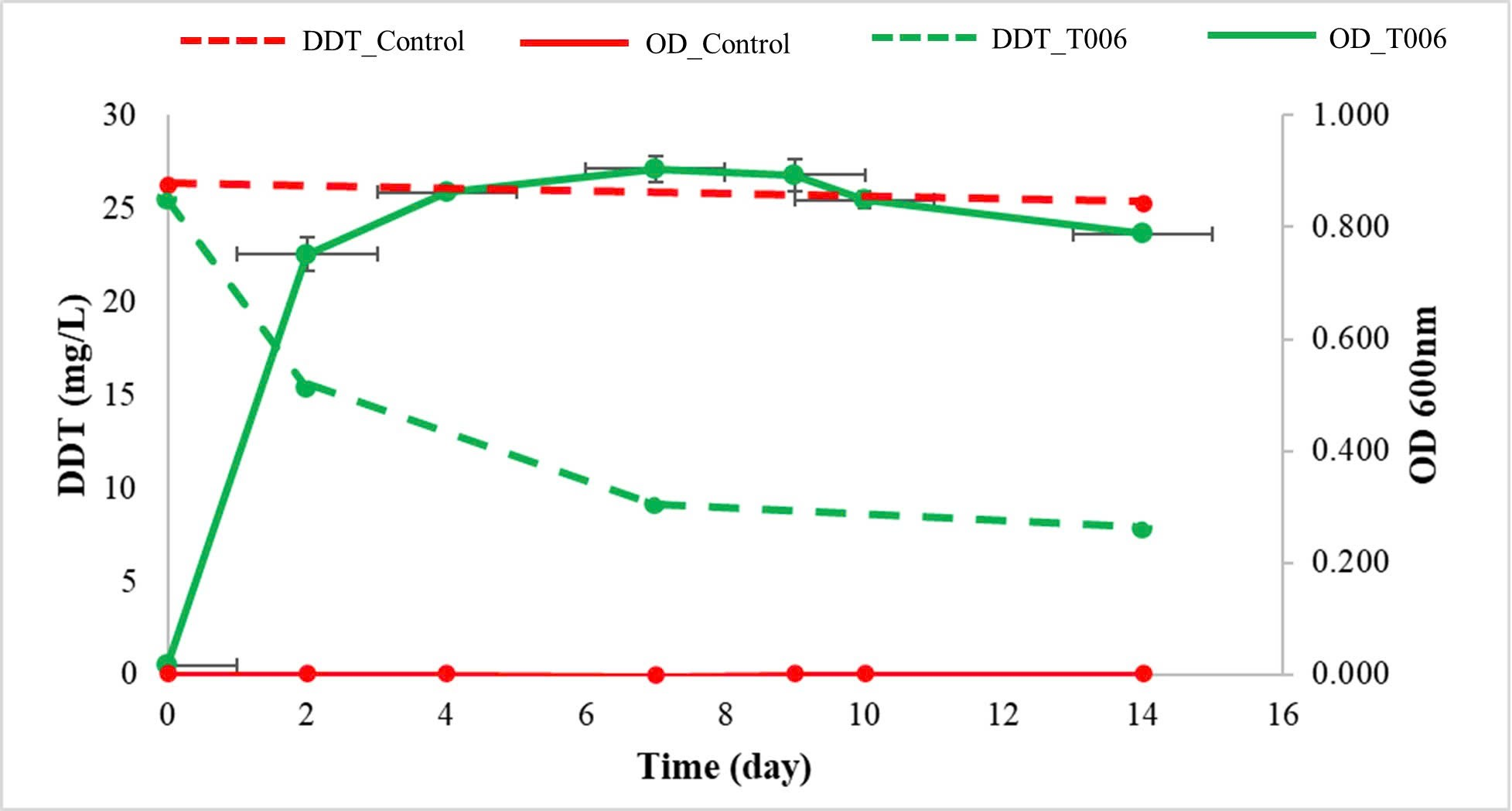


Figure S1. DDT degradation and growth kinetics of T006 when tested in a liquid medium initially containing 30 mg L-1 DDT. Note: Control: no bacterial cells.

**Monitoring metabolites during DDT degradation**

DDT and possible intermediates were monitored during a 2-week period of degradation experiment in liquid medium as described above. Specifically, the samples taken at day 0, day 2, day 7 and day 14 were also subjected to GC-MS to analyze the metabolites. The detailed analysis procedure is as follows:

Each sample was extracted with chloroform at a volumetric ratio of 1:1 (sample:chloroform), vortexed for 5 min, and the organic phase was collected. The extracts were analyzed using a gas chromatograph (Agilent 7890) equipped with a glass capillary HP-5MS column (ID 0.25 mm, length 30 m, film thickness 0.25 µm; part no. 19091S-433). Helium was used as the carrier gas at a flow rate of 1.0 mL min⁻¹. The injector was operated in split mode (1:20) with an injection volume of 1 µL.

The injector temperature was set at 280 °C. The oven temperature program was as follows: initial temperature 80 °C held for 1 min, ramped at 40 °C min⁻¹ to 300 °C and held for 1.7 min, followed by a post-run at 300°C for 0.5 min. The GC/MSD 5977C system was operated under the following conditions: transfer line temperature 300°C, ion source temperature 230°C, quadrupole temperature 150°C, and solvent delay 4.5 min. Concentrations of DDT and its transformation products were quantified using MassHunter software, and compound identification was performed by comparison with the NIST mass spectral library.

The results (Figure S2) showed that only DDE could be detected at significant amount after around 7 days of degradation but its concentration also decreased further to an insignificant level after 14 days. These results suggest that the degradation is not only transformation but includes several steps that may further lead to complete mineralization.


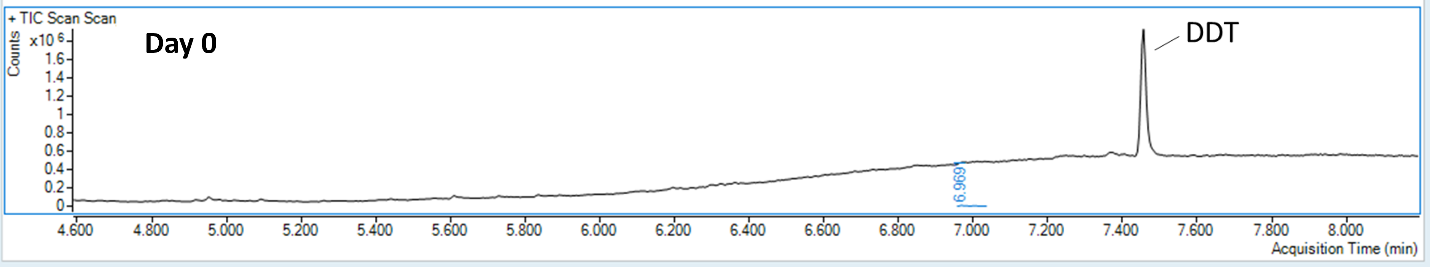


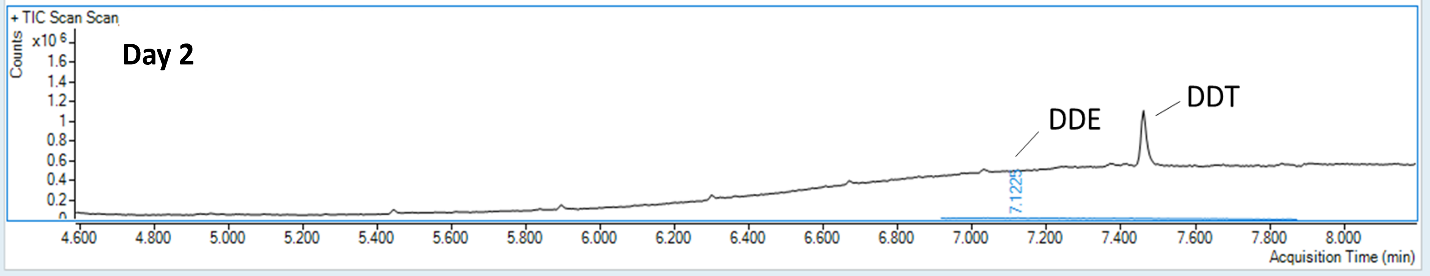


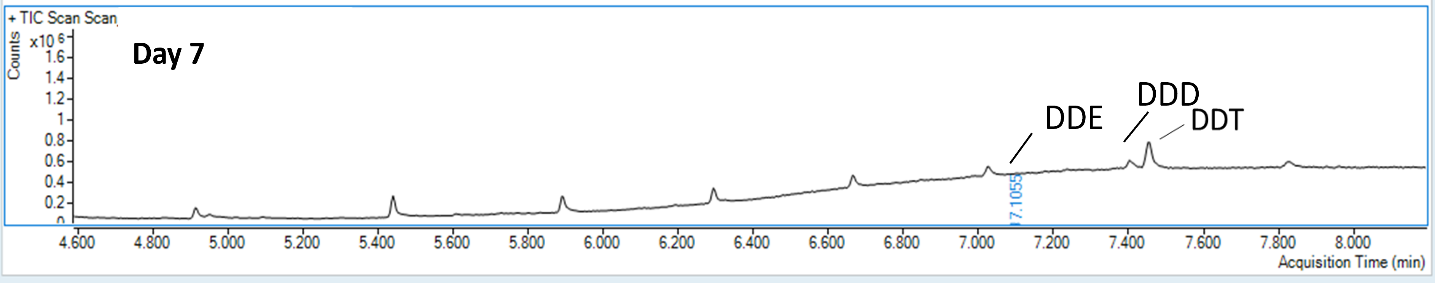


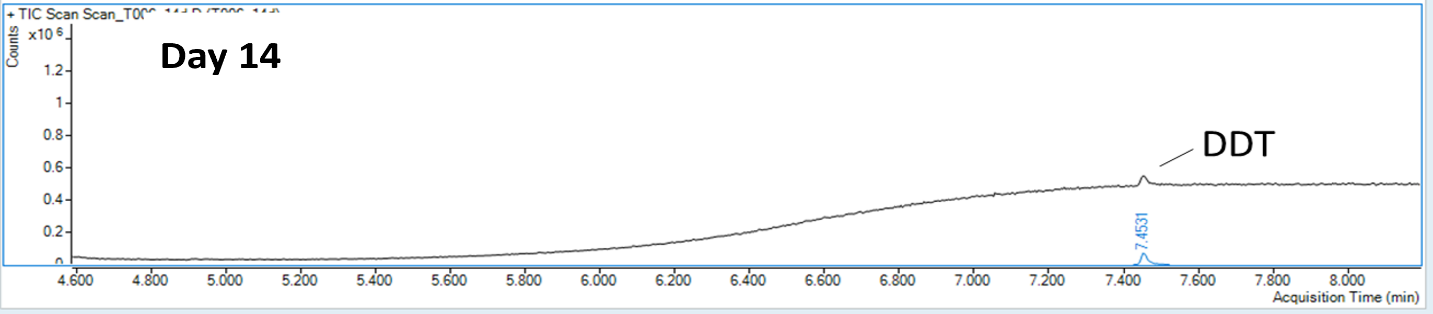


Figure S2. GS-MS profiles of the metabolites in the liquid culture of T006 during a 2-week period experiment testing its DDT degradation.

Table S3. DDT-degrading performance of *Pseudomonas vietnamensis* T006 in comparison with those of some other known DDT degrading bacteria

| **Microorganism** | **DDT** | **Biodegradation (%)** | **Time** | **References** |
| --- | --- | --- | --- | --- |
| ***Pseudomonas vietnamensis* T006** | 30mg/L | > 70%  > 60% | 8W  7d |  |
| *Aeromonas hydrophila* | 14–15 µM | 73 | 20d | Cao, F., Liu, T. X., Wu, C. Y., Li, F. B., Li, X. M., Yu, H. Y., Tong, H., & Chen, M. J. (2012). Enhanced biotransformation of DDTs by an iron- and humic-reducing bacteria Aeromonas hydrophila HS01 upon addition of goethite and anthraquinone-2,6-disulphonic disodium salt (AQDS). Journal of Agricultural and Food Chemistry., 60, 11238–11244. |
| *Chryseobacterium* sp. PYR2 | 50 mg/L | 40 | 4-6d | Qu, J., Xu, Y., Ai, G. M., Liu, Y., & Liu, Z. P. (2015). Novel Chryseobacterium sp. PYR2 degrades various organochlorine pesticides (OCPs) and achieves enhancing removal and complete degradation of DDT in highly contaminated soil. Journal of Environmental Management., 161, 350–357. |
| *Pseudoxanthomonas* sp. | 100mg/L | 60 | 144h | Wang, G., Zhang, J., Wang, L., Liang, B., Chen, K., Li, S., & Jiang, J. (2010). Co-metabolism of DDT by the newly isolated bacterium Pseudoxanthomonas sp. Brazilian Journal of Microbiology., 41, 341–338. |
| *Rhodococcus* sp. strain IITR03 | 50mg/L | 75 | 21d | Fang, H., Dong, B., Yan, H., Tang, F., & Yu, Y. (2010). Characterization of a bacterial strain capable of degrading DDT congeners and its use in bioremediation of contaminated soil. Journal Hazardous Material., 184, 281–289 |
| *Sphingobacterium* sp. | 25ppm | 28.48 | 144h | Bidlan, R., & Manonmani, H. K. (2002). Aerobic degradation of dichlorodiphenyltrichloroethane (DDT) by Serratia marcescens DT-1P. Process Biochemestry., 38, 49–56 |
| *Staphylococcus* sp. | 100ppm | 69 | 30d | Mwangi, K., Boga, H. I., Muigai, A. W., Kiiyuikia, C., & Tsanuo, M. K. (2010). Degradation of dichlorodiphenyltrichloroethane (DDT) by bacterial isolates from cultivated and uncultivated soil. African Journal of Microbiology Research., 4, 185–196 |
| *Stenotrophomonas* sp. DDT-1 | 10mg/L | 100 | 7d | Pan, X., Lin, D., Zheng, Y., Zhang, Q., Yin, Y., Cai, L., Fang, H., & Yu, Y. (2016). Biodegradation of DDT by Stenotrophomonas sp. DDT-1: characterization and genome functional analysis. Scientific Reports, 6, 21332. |

**Additional information regarding the profile of strain T006:**

**16S RNA sequence:**

AAAGGAACGCTAATACCGCATACGTCCTACGGGAGAAAGCAGGGGACCTTCGGGCCTTGCGCTATCAGATGAGCCTAGGTCGGATTAGCTAGTTGGTGGGGTAAAGGCCTACCAAGGCGACGATCCGTAACTGGTCTGAGAGGATGATCAGTCACACTGGAACTGAGACACGGTCCAGACTCCTACGGGAGGCAGCAGTGGGGAATATTGGACAATGGGCGAAAGCCTGATCCAGCCATGCCGCGTGTGTGAAGAAGGTCTTCGGATTGTAAAGCACTTTAAGTTGGGAGGAAGGGCAGTAAGTTAATACCTTGCTGTTTTGACGTTACCAACAGAATAAGCACCGGCTAACTTCGTGCCAGCAGCCGCGGTAATACGAAGGGTGCAAGCGTTAATCGGAATTACTGGGCGTAAAGCGCGCGTAGGTGGTTTGGTAAGATGGATGTGAAATCCCCGGGCTCAACCTGGGAACTGCATCCATAACTGCCTGACTAGAGTACGGTAGAGGGTGGTGGAATTTCCTGTGTAGCGGTGAAATGCGTAGATATAGGAAGGAACACCAGTGGCGAAGGCGACCACCTGGACTGATACTGACACTGAGGTGCGAAAGCGTGGGGAGCAAACAGGATTAGATACCCTGGTAGTCCACGCCGTAAACGATGTCGACTAGCCGTTGGGATCCTTGAGATCTTAGTGGCGCAGCTAACGCGATAAGTCGACCGCCTGGGGAGTACGGCCGCAAGGTTAAAACTCAAATGAATTGACGGGGGCCCGCACAAGCGGTGGAGCATGTGGTTTAATTCCAACCAACCCAAAAAACCTTACCTGGCCTTGACATGTCCGGAATCTTGCAGAGATGCGAGAGTGCCTTCGGGAATCGGAACACAGGTGCTGCATGGCTGTCGTCAGCTCGTGTCGTGAGATGTTGGGTTAAGTCCCGTAACGAGCGCAACCCTTGTCCTTAGTTACCAGCACGTTATGGTGGGCACTCTAAGGAGACTGCCGGTGACAAACCGGAGGAAGGTGGGGATGACGTCAAGTCATCATGGCCCTTACGGCCAGGGCTACACACGTGCTACAATGGTCGGTACAGAGGGTTGCCAAGCCGCGAGGTGGAGCTAATCCCATAAAACCGATCGT

**Housekeeping gene (*rpoD*) sequence:**

TAGACGGCATCCTCGAAGAGTACAACCGTGTGGTTGCCGAAGGCGGCCGCCTGTCGGACATCCTCAGCGGCTACATCGACCCGGATGACGGCAGCCTGCCGGCCGAGGAAGTTGAGCCGGTCGGTCTGAAAGACGACGCCGAAGCCAAGGAAAAGGACGAAGAGGACGAAGAGTCCGACGGCGATAGCGAAGAAGAAGAAGGCGACGGTGGTCCGGATCCGGAAGAAGCCGCTCGTCGTTTCGGCGCCGTTTCCGAGCAACTGGAAAAGGCCAAGAAAGCCCTGAAGAAGCACGGCCGCGGCAGCAAGCAGGCCAGCGAGGAACTGCTCGCTCTGGCCGAACTGTTCATGCCGATCAAGCTCGTGCCCAAGCAGTTCGACGTGCTGGTCGCCAAGGTCCGCGATTCGCTGGACAGCGTTCGCCGCCAGGAACGCGCCATCATGCAGCTGTGCGTGCGTGATGCCCGCATGCCGCGTGCCGACTTCCTGCGCCAGTTCCCCGGCCACGAAACCGACACCGCCTGGGTCGACGCCGTACTCAAGGGCAAGCCCAAGTACGCCGAGGCCATCGAGCGCCTG

**Table P1.** Effect of temperature on the Cl⁻ removal rate

|  | **Rate of chlorine removal (mM week⁻¹) after 1 week** | | | | |
| --- | --- | --- | --- | --- | --- |
|  | 10^o^C | 15^o^C | 25^o^C | 30^o^C | 40^o^C |
| T006 | 0.096±0.025 | 0.267±0.016 | 0.195±0.063 | 0.425±0.145 | 0.351±0.024 |

**Table P2.** Effect of pH on the Cl⁻ removal rate

| Strain | **Rate of chlorine removal (mM week⁻¹) after 1 week** | | | | |
| --- | --- | --- | --- | --- | --- |
|  | pH3 | pH5 | pH7 | pH9 | pH11 |
| T006 | 0.328±0.025 | 0.456±0.016 | 0.425±0.145 | 0.512±0.027 | 0.478±0.072 |

**Table P3.** Effect of DDT concentration on the Cl⁻ removal rate

|  | **Rate of chlorine removal (mM week⁻¹) after 1 week** | | | | |
| --- | --- | --- | --- | --- | --- |
|  | 2ppm | 5ppm | 10ppm | 20ppm | 30ppm |
| T006 | 6.305±0.009 | 4.998±0.141 | 4.071±0.499 | 1.986±0.379 | 0.414±0.121 |

**Table P4.** Effect of carbon sources on the Cl⁻ removal rate

|  | **Rate of chlorine removal (mM week⁻¹) after 1 week** | | | | | |
| --- | --- | --- | --- | --- | --- | --- |
|  | Fructose | Galactose | Maltose | Lactose | Sucrose | Phenol |
| T006 | 0.436±0.036 | 0.383±0.033 | 0.398±0.055 | 0.14±0.046 | 0.329±0.039 | 0.16±0.008 |

**Table P5.** Effect of nitrogen sources on the Cl⁻ removal rate

|  | **Rate of chlorine removal (mM week⁻¹) after 1 week** | | | | |
| --- | --- | --- | --- | --- | --- |
|  | Amonium citracte | Amonium sunfate | Potassium Nitrate | Peptone | Glycine |
| T006 | 0.318±0.095 | 0.193±0.045 | 0.24±0.115 | 0.47±0.094 | 0.03±0.007 |

**Table P6.** Effect of TE on the Cl⁻ removal rate

|  | **Rate of chlorine removal (mM week⁻¹) after 1 week** | | | | |
| --- | --- | --- | --- | --- | --- |
|  | 0,1% | 0,5% | 1% | 2% | 5% |
| T006 | 0.189±0.067 | 0.227±0.05 | 0.135±0.009 | 0.161±0.014 | 0.05±0.005 |

**Table P7.** Effect of inoculum size on the Cl⁻ removal rate

|  | **Rate of chlorine removal (mM week⁻¹) after 1 week** | | | |
| --- | --- | --- | --- | --- |
|  | 5% | 10% | 15% | 20% |
| T006 | 0.21±0.072 | 0.414±0.074 | 0.425±0.051 | 0.493±0.151 |

**2.2. Physiological characteristics:**

Mobility:

| T006 | T069 ((+)control) | *Staphylococcus* sp.  ((-) control) |
| --- | --- | --- |
| 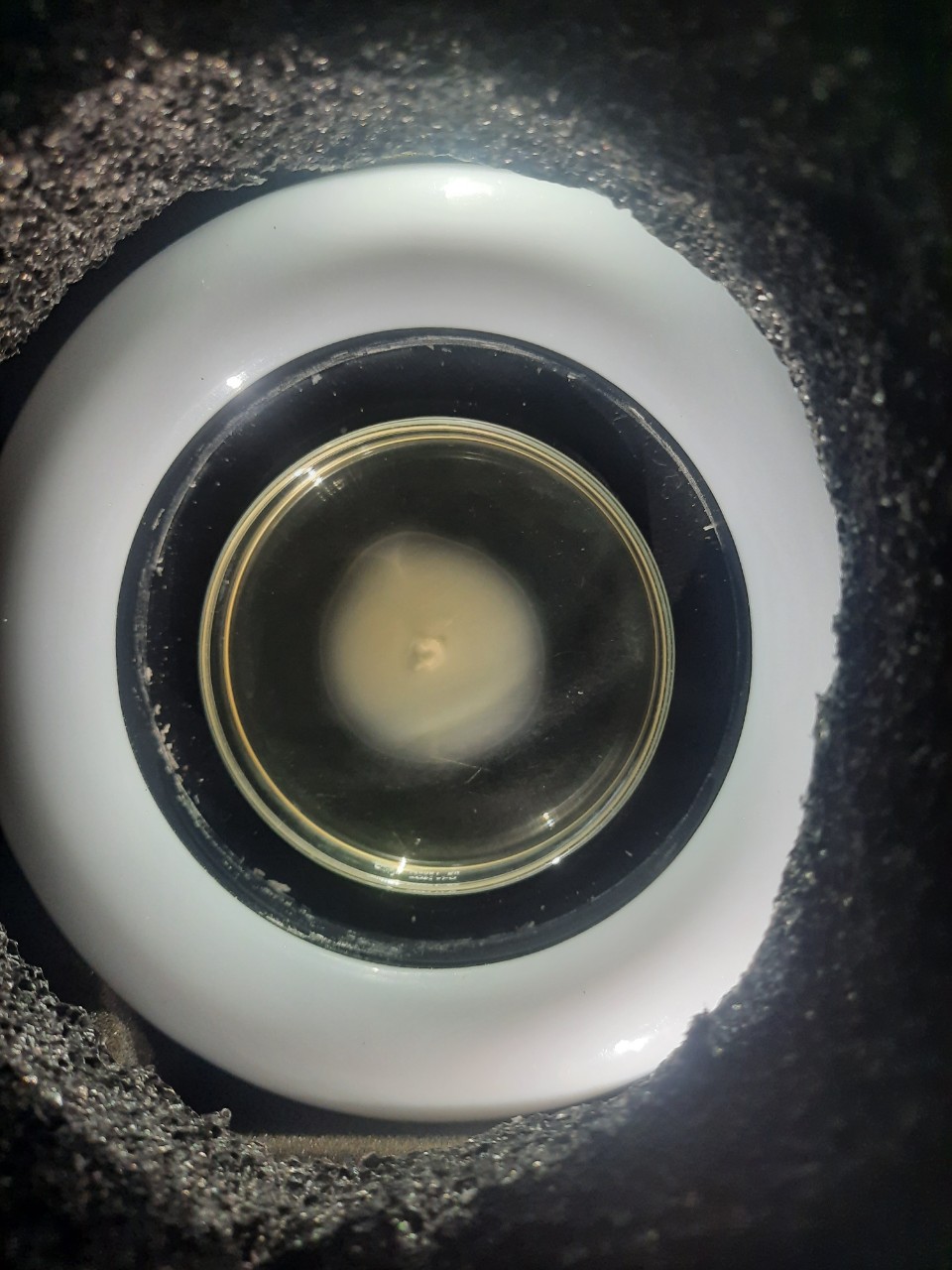 | 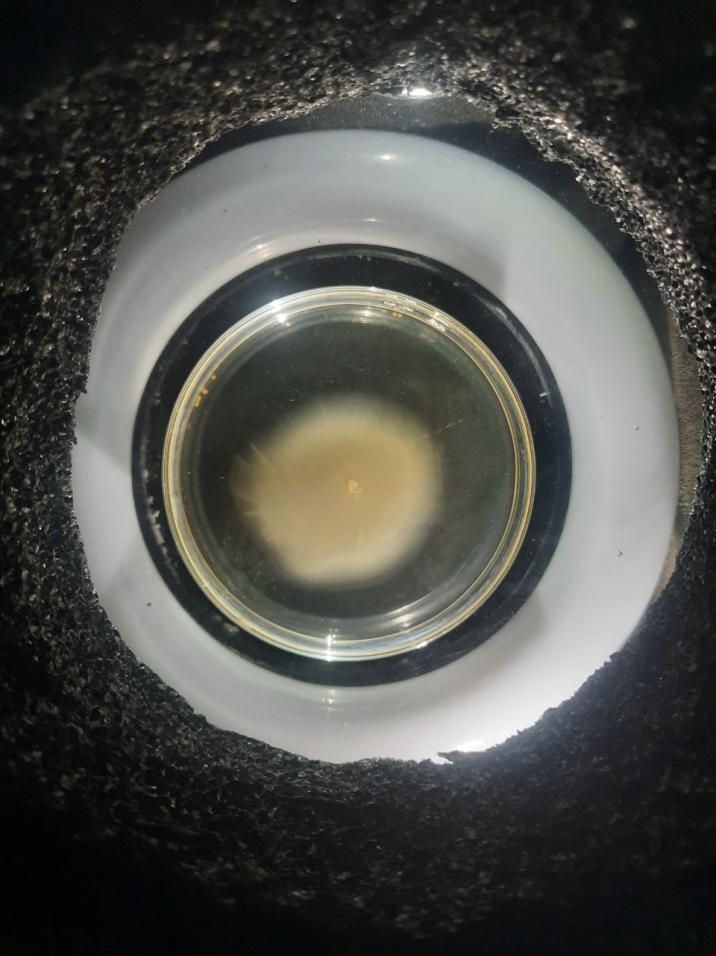 | 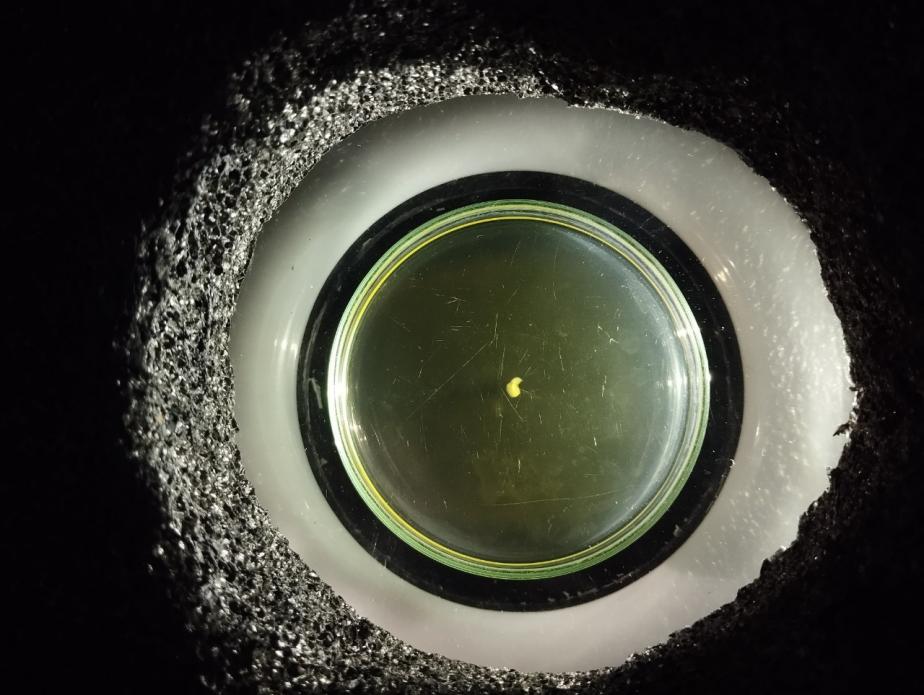 |

**2.3. Biochemical characteristics:**

**a. Bioluminescene:**

| T006 | PAM68 (*Pseudomona fluorescens*) ((+)control) | PAM64 (*Pseudomonas lalkuanensis*)((-) control) |
| --- | --- | --- |
| 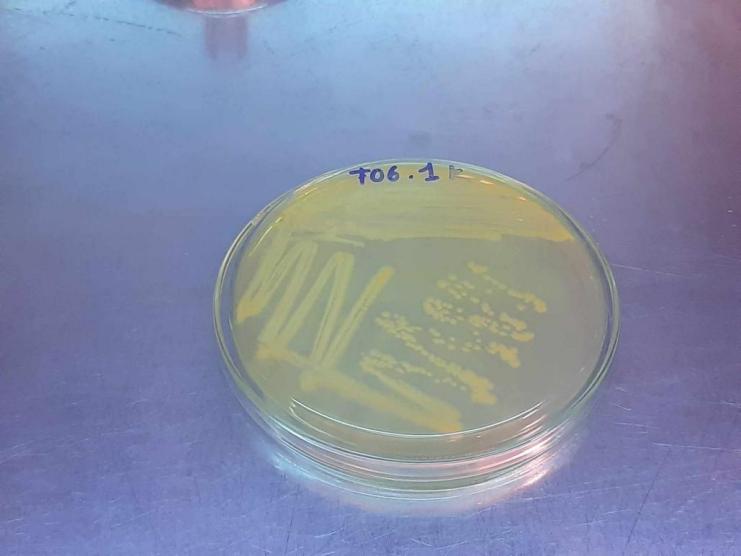 | 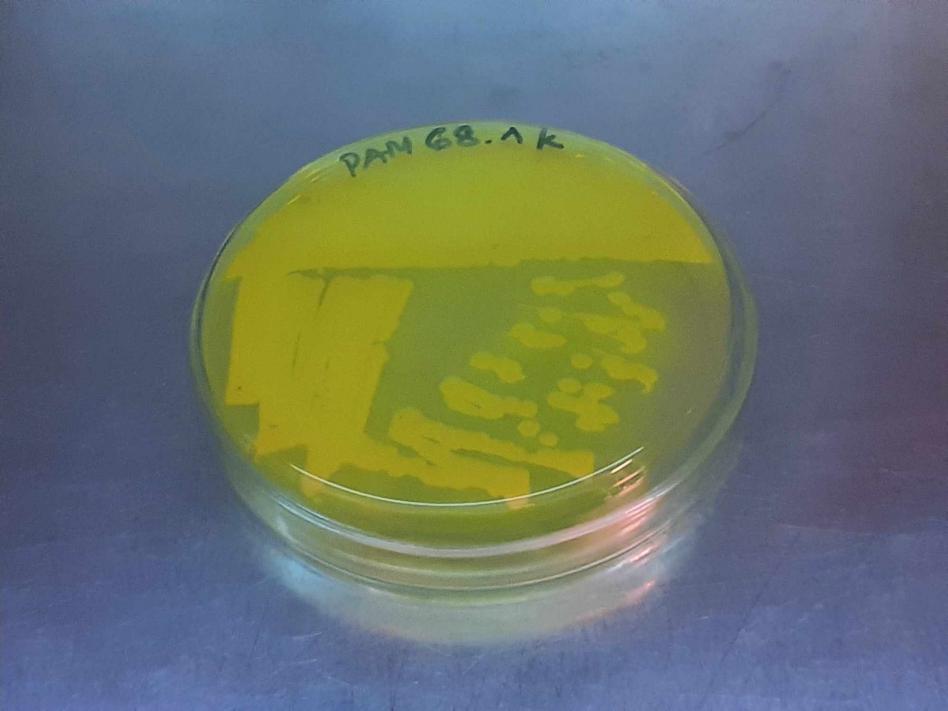 | 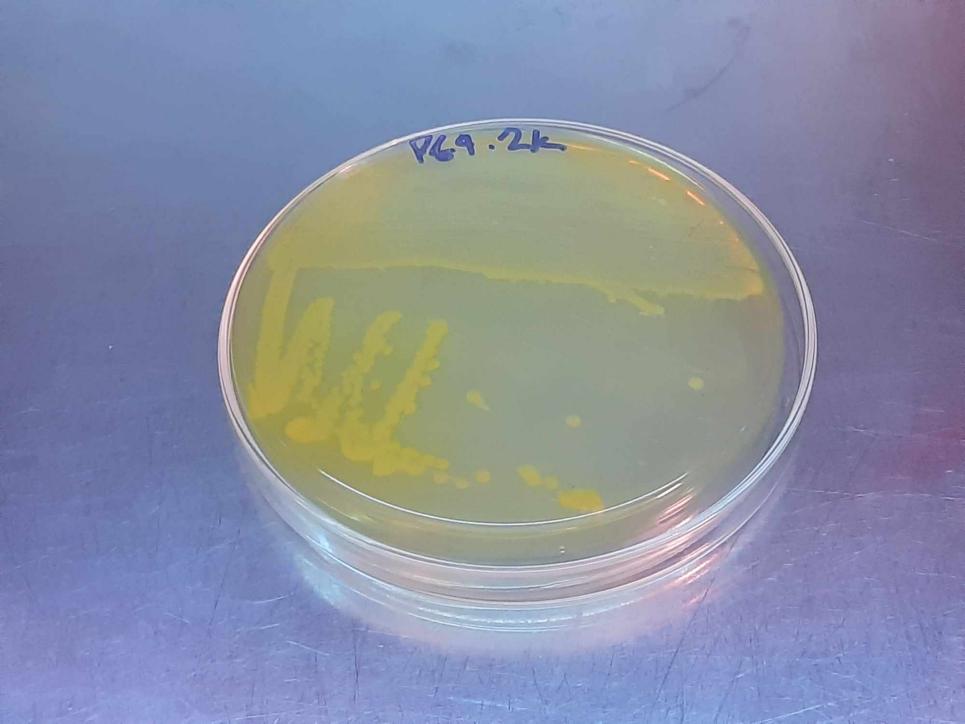 |

**b. Kit 20NE**

* Biochemical results according to API20NE Kit:

**
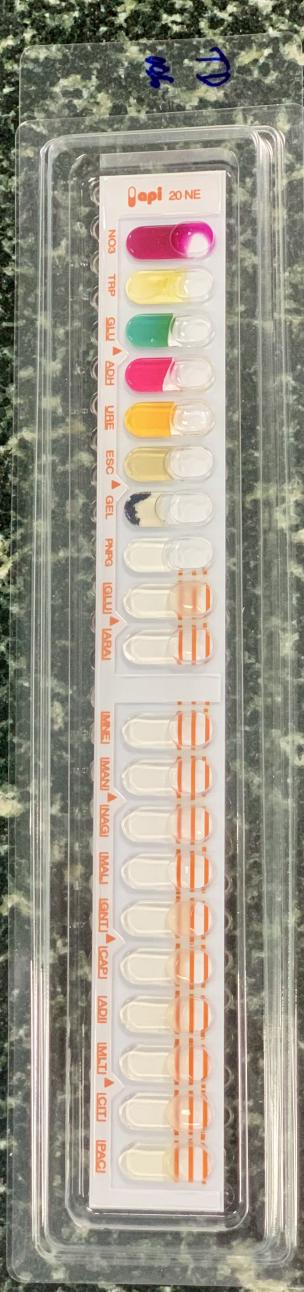
**

| **NO3** | **TRP** | **GLU** | **ADH** | **URE** | **ESC** | **GEL** | **PNPG** | **GLU** | **ARA** |
| --- | --- | --- | --- | --- | --- | --- | --- | --- | --- |
| **+** | **-** | **-** | **+** | **+** | **-** | **-** | **-** | **+** | **-** |
| **MNE** | **MAN** | **NAG** | **MAL** | **GNT** | **CAP** | **ADI** | **MLT** | **CIT** | **PAC** |
| **-** | **-** | **-** | **-** | **+** | **+** | **+** | **+** | **+** | **+** |
